# Molecular Cloning and Functional Characterization of Mannose Receptor in Zebra Fish (*Danio rerio*) during Infection with *Aeromonas sobria*

**DOI:** 10.3390/ijms160510997

**Published:** 2015-05-15

**Authors:** Feifei Zheng, Muhammad Asim, Jiangfeng Lan, Lijuan Zhao, Shun Wei, Nan Chen, Xiaoling Liu, Yang Zhou, Li Lin

**Affiliations:** 1 Department of Aquatic Animal Medicine, College of Fisheries, Huazhong Agricultural University, Wuhan 430070, China; E-Mails: zhengfeifei@webmail.hzau.edu.cn (F.Z.); asim_m97@yahoo.com (M.A.); lanjiangfeng@mail.hzau.edu.cn (J.L.); zhaolijuan4234@163.com (L.Z.); weishun@mail.hzau.edu.cn (S.W.); chennan@mail.hzau.edu.cn (N.C.); liuxl@mail.hzau.edu.cn (X.L.); 2 Freshwater Aquaculture Collaborative Innovation Center of Hubei Province, Wuhan 430070, China; 3 Key Lab of Freshwater Animal Breeding, Ministry of Agriculture, Wuhan 430070, China; 4 Agricultural Bioinformatics Key Laboratory of Hubei Province, College of Informatics, Huazhong Agricultural University, Wuhan 430070, China

**Keywords:** *Danio rerio*, mannose receptor, mRNA expression, protein expression, *Aeromonas sobria*

## Abstract

Mannose receptor (MR) is a member of pattern-recognition receptors (PRRs), which plays a significant role in immunity responses. Much work on MR has been done in mammals and birds while little in fish. In this report, a MR gene (designated as zfMR) was cloned from zebra fish (*Danio rerio*), which is an attractive model for the studies of animal diseases. The full-length cDNA of zfMR contains 6248 bp encoding a putative protein of 1428 amino acids. The predicted amino acid sequences showed that zfMR contained a cysteine-rich domain, a single fibronectin type II (FN II) domain, eight C-type lectin-like domains (CTLDs), a transmembrane domain and a short *C*-terminal cytoplasmic domain, sharing highly conserved structures with MRs from the other species. The MR mRNA could be detected in all examined tissues with highest level in kidney. The temporal expression patterns of MR, IL-1β and TNF-α mRNAs were analyzed in the liver, spleen, kidney and intestine post of infection with *Aeromonas sobria*. By immunohistochemistry assay, slight enhancement of MR protein was also observed in the spleen and intestine of the infected zebra fish. The established zebra fish-*A. sobria* infection model will be valuable for elucidating the role of MR in fish immune responses to infection.

## 1. Introduction

Mannose receptor (MR) is one of the pattern-recognition receptors (PRRs), which play a significant role in innate immunity responses through binding to the pathogen-associated molecular patterns (PAMPs) [1]. MR primarily expresses in macrophages and dendritic cells and is a member of the C-type lectin superfamily [2]. The structure of MR contains extracellular, transmembrane and cytoplasmic regions. In the extracellular region, it consists of three domains: an *N*-terminal cysteine-rich (CR) domain, a fibronectin type II (FN II) domain and eight tandemly arranged C-type lectin-like domains (CTLDs). MR recognizes surface polysaccharides of various pathogens, such as viruses, bacteria, yeasts and parasites, including HIV [3], Dengue virus [4], *Candida albicans* [5], *Mycobacterium tuberculosis* [6,7], *Pneumocystis carinii* [8,9], *Cryptococcus neoformans* [10], *Klebsiella pneumoniae* [11], *Streptococcus pneumoniae* [11] and *Leishmania* [12,13], *etc.* In recruited inflammatory peritoneal macrophages, MR levels were increased in response to interleukin-4 (IL-4), IL-13 and IL-10 [14]. Due to the critical role in innate immunity responses, there have been many reports about MR in human and mice.

Zebra fish (*Danio rerio*) has become an attractive research model for the studies of human and fish diseases [15]. *Aeromonas* is a genus of the family *Enterobacteriaceae*. Most species in *Aeromonas* are pathogens of animals. *Aeromonas sobria* can cause serious diseases of both fish and human [16,17,18,19,20,21,22]. Also bacteria infection can experimentally cause immune responses in fish [23]. Therefore, the establishment of a zebra fish-*A. sobria* infection model will be important for the studies of fish as well as human diseases. We have isolated a strain of *A. sobria* which could cause the disease of zebra fish in our lab. We have focused our studies on the functions of teleost MRs [24,25,26,27]. In the present report, we cloned and characterized the functions of zebra fish MR using the established zebra fish-*A. sobria* infection model. The results will provide an insight into the understanding of the functions of teleost MRs.

## 2. Results

### 2.1. Characterization of zfMR cDNA

**Figure 1:**
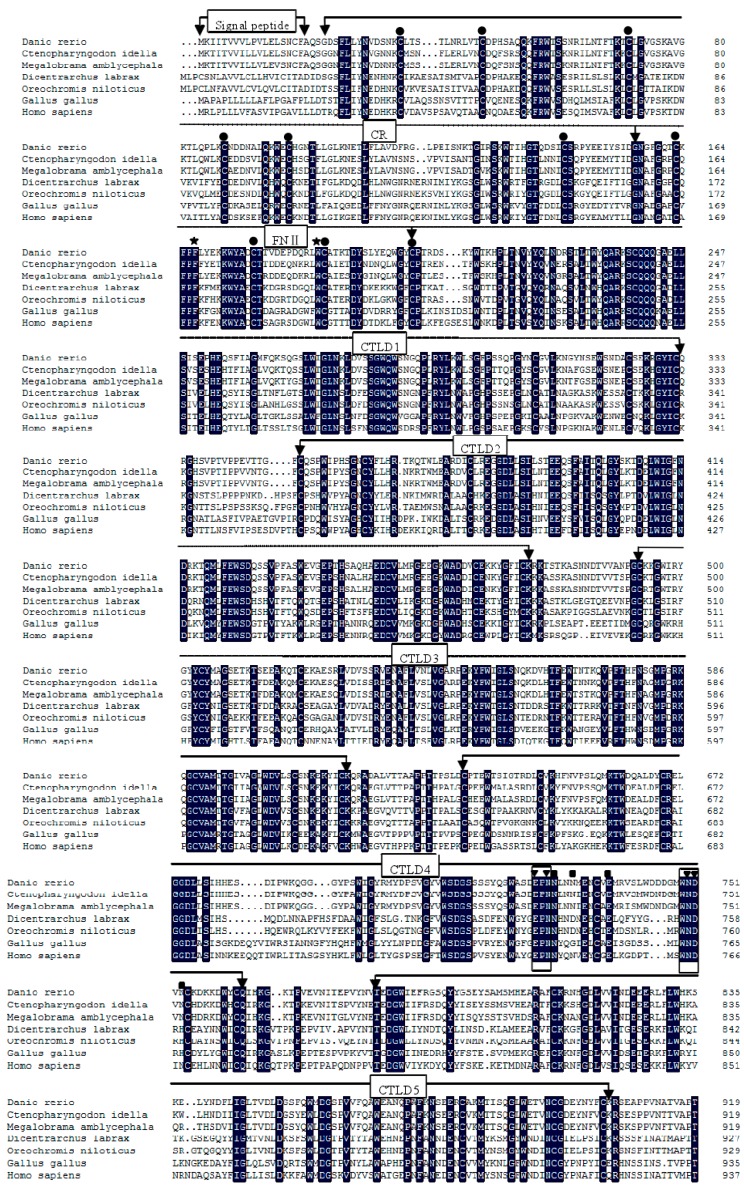

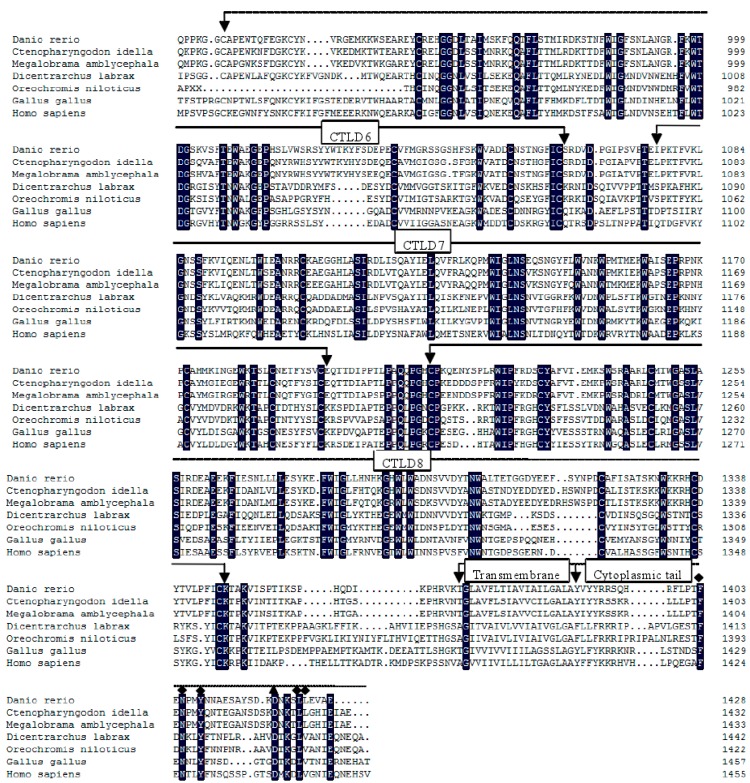
Multiple alignment of mannose receptor (MRs) using the DNAMAN program. The identical residues among all the MRs were in black. The absent amino acids in the alignment were indicated by dots (.). Conserved cysteine residues were marked with ●, aromatic residues F (Phe^167^) and W (Trp^188^) were marked with ★. Two conserved sites of carbohydrate recognition domain (CRD), “EPN” and “WND” were boxed. Ca^2+^ binding site 1 was marked with ■, Ca^2+^ binding site 2 was marked with ▼. Two potential endocytosis motifs, “F-N-Y” and a dihydrophobic motif “LL” were marked with ♦. The conserved acidic residue “D” lying-4 to the dihydrophobic modif was marked with ▲. The GenBank accession numbers of the MRs were as follows: *Danio rerio* (KP172154); *Ctenopharyngodon idella* (KF569903.1); *Megalobrama amblycephala* (KC495437.1); *Dicentrarchus labrax* (CBN82067.1); *Oreochromis niloticus* (XP_003439398.1); *Gallus gallus* (XP_004939311.1); *Homo sapiens* (NM_002438.2).

The full-length cDNA of zfMR was deposited in GenBank (accession number KP172154). The complete sequences of MR cDNA consisted of a 5'terminal untranslated region (UTR) of 119 bp, a 3'UTR of 1842 bp with a poly (A) tail, and an open reading frame (ORF) of 4287 bp (Supplementary Figure S1). The ORF encoded a polypeptide of 1428 amino acids, including a 19 aa signal peptide with an isoelectric point of 6.15 and predicted molecular weight of 163.2 kD. The typical polyadenylation signal AATAAA was found 18 bp before the poly A signal (Supplementary Figure S1). As shown in Figure 1, zfMR contained an *N*-terminal cysteine-rich (CR) domain (22–156 aa), a fibronectin type II (FN II) domain (156–204 aa) and eight tandemly arranged C-type lectin-like domains (CTLDs) (204–333, 350–474, 493–615, 635–763, 783–905, 926–1062, 1076–1194, 1211–1347 aa), a transmembrane domain (1370–1392 aa) and a short cytoplasmic tail (1392–1428 aa). The CR domain contained six conserved cysteines, which were responsible for the formation of three disulfide bonds. The fibronectin type II contained four conserved cysteines and formed two disulfide bonds [2]. The CTLDs possessed two conserved carbohydrate recognition sites, EPN (Glu^725^-Pro^726^-Asn^727^) and WND (Trp^749^-Asn^750^-Asp^751^) [28,29]. In addition, there were two conserved Ca^2+^ binding sites (Asn^728^-Asn^731^-Glu^737^-Asn^753^ and Glu^725^-Asn^727^-Asn^750^-Asp^751^) [2]. The cytoplasmic domain contained two potential endocytosis motifs with (F^1403^-N^1405^-Y^1408^) based on a conserved tyrosine residue (Y^1408^) and a dihydrophobic motif (L^1423^L^1424^) [2].

### 2.2. Phylogenetic Relationship of MRs

**Figure 2:**
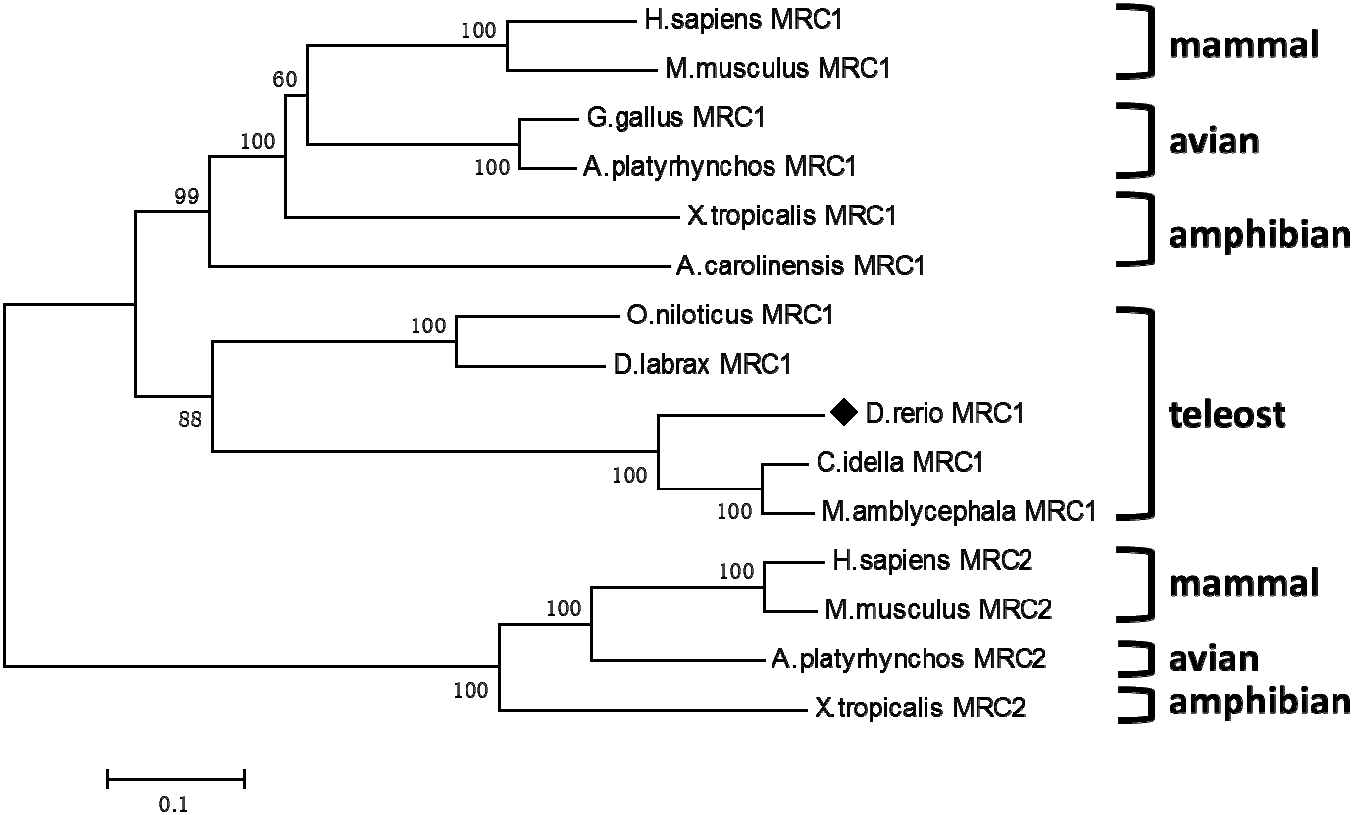
Phylogenetic tree of MR from *Danio rerio* (KP172154) and other species were constructed using Molecular Evolutionary Genetics Analysis (MEGA) 4.0 with neighbor-joining method. Numbers of each node indicated the percentage of bootstrapping of a 1000 replications. *Danio rerio* MRC1 (KP172154) was marked with ♦. The protein sequences used for phylogenetic analysis were *Anolis carolinensis* MRC1 (XM_003222081.1), *Xenopus tropicalis* MRC1 (XM_002939002.2), *Anas platyrhynchos MRC1* (EOB05991.1), *Mus musculus* MRC1 (NM_008625.2), *Homo sapiens* MRC2 (NM_006039.4), *Mus musculus* MRC2 (NM_008626.3), *Anas platyrhynchos* MRC2 (XM_005030646.1), *Xenopus tropicalis* MRC2 (NM_001097247.1), other sequences were shown in the legend of Figure 1.

Based on the multiple alignment of MRs from nine taxa including three teleosts, two amphibians, two avians and two mammals. The phylogenetic tree clearly showed that the MR genes formed teleost, amphibian, avian and mammal clades. MRs from zebra fish, grass carp (*Ctenopharyngodon idella*) and blunt snout bream (*Megalobrama amblycephala*) were clustered in teleost clade. Finally, the clade from fish was clustered with amphibian, avian and mammal clades successively (Figure 2).

### 2.3. The mRNA Expression Profile of MR in Different Tissues

**Figure 3:**
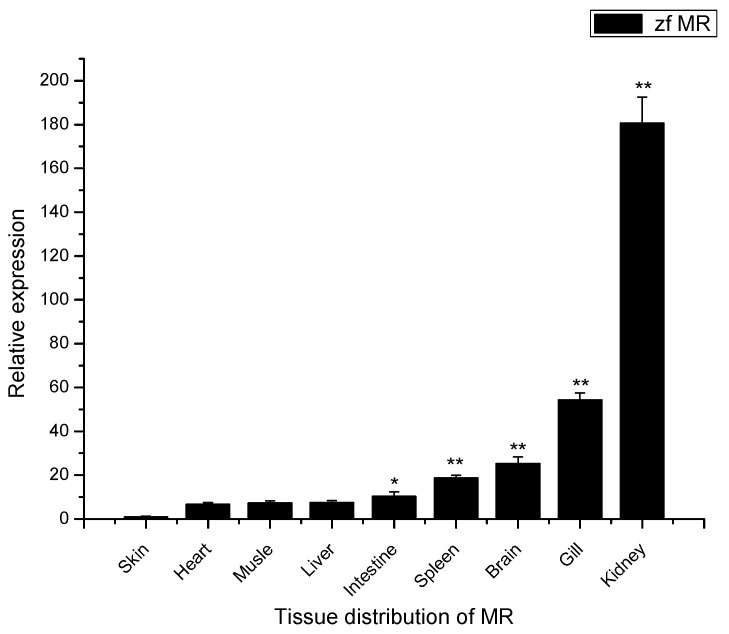
Expression analysis of MR mRNAs in various tissues of zebra fish by Quantitative Real-Time PCR (qRT-PCR). The expression level of MR in the skin was set as 1, expression levels in all other tissues were presented relative to that in the skin. Expression of β-actin was used as an internal control for qRT-PCR. Each experiment was performed in triplicate. Data were shown as mean ± SE (*n* = 3). The asterisk indicated a statistically significant difference (** *p* < 0.01, * *p* < 0.05) compared with skin (set as 1).

In zebra fish, the mRNA transcripts of MR were found to be constitutively expressed in a wide range of tissues with different levels, including liver, spleen, kidney, intestine, gill, brain, heart, muscle and skin (Figure 3). The lowest level MR mRNA was detected in skin. To compare the relative expression of the mRNA in different tissues, the MR transcript levels in other tissues were normalized to that in skin (set as 1). The highest level MR transcript was in kidney followed by gill, brain, spleen, intestine, liver, muscle, and heart, respectively. The amount of zfMR mRNAs in each of kidney, gill, brain, spleen, and intestine was all significantly higher than that in skin (Figure 3).

### 2.4. Temporal Expression of MR after Infection with A. sobria

**Figure 4:**
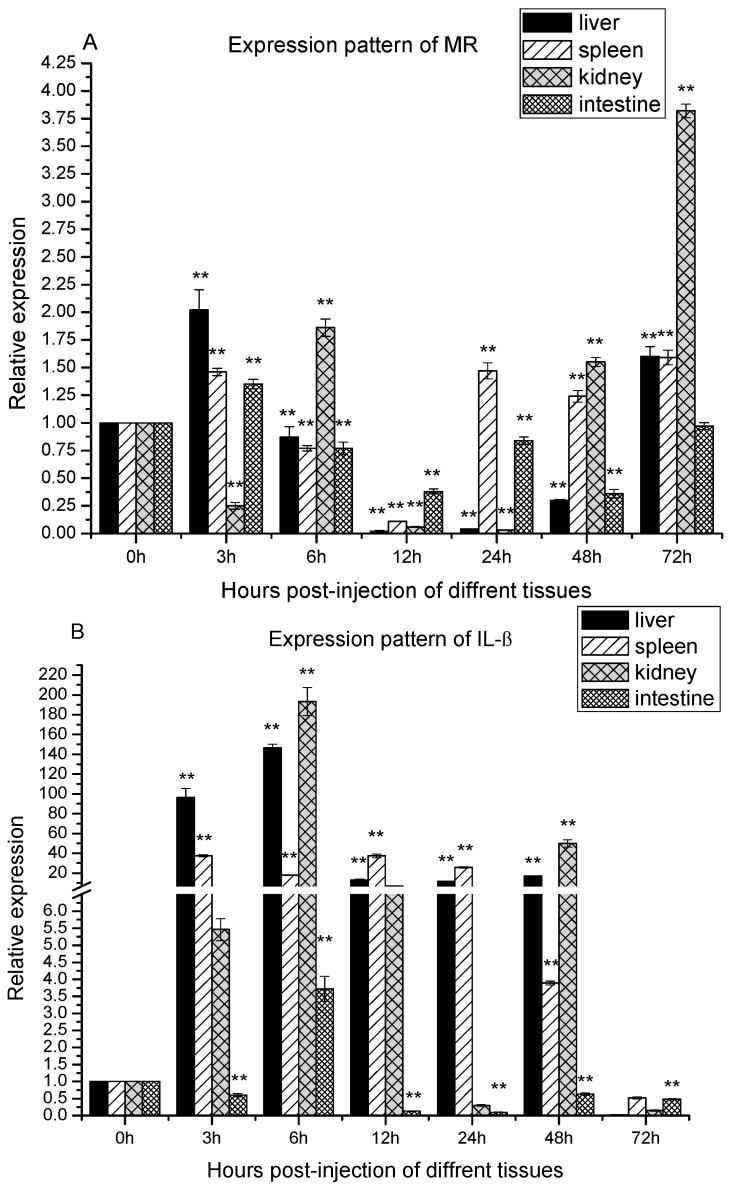

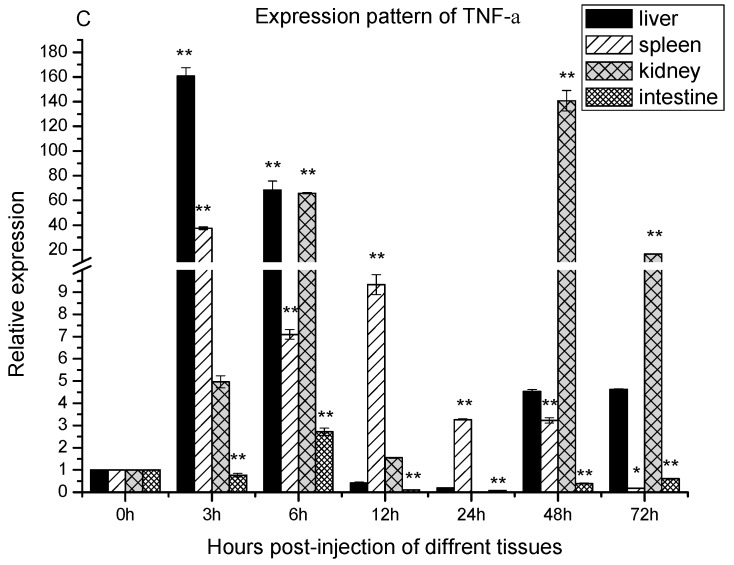
The expression of MR, interleukin-1β (IL-1β) and TNF-α after *Aeromonas sobria* infection. Expression patterns of MR (**A**); IL-1β (**B**) and TNF-α (**C**) were determined in liver, spleen, kidney and intestine by qRT-PCR. The samples were analyzed at 0, 3, 6, 12, 24, 48 and 72 h post-injection. Expression of β-actin was used as internal control for qRT-PCR. Each experiment was performed in triplicate. Data were shown as mean ± SE (*n* = 3). The asterisk indicated a statistically significant difference (** *p* < 0.01, * *p* < 0.05) compared with 0 h (set as 1).

In order to confirm that the zebra fish MR was involved in response to *A. sobria* challenge, qRT-PCR was performed to detect the MR mRNA in the liver, kidney, spleen and intestine which had a close relationship with immune responses. As shown in Figure 4A, there were basically one-peak and two-peak expression patterns of MR in the four tissues during the infection. In the liver, spleen and kidney, the MR expression fits a two-peaks pattern. In the liver, it was significantly up-regulated at 3 h poi. Subsequently, significantly, it dropped under the basal level until 48 h. The second peak was observed at 72 h poi. In the case of the spleen, the MR gene expression pattern was similar to that in liver except the second peak came up early at 24 h poi. In the case of the kidney, it first significantly dropped at 3 h poi, then the first peak came up at 6 h poi. Subsequently, it decreased until 24 h poi and it increased significantly during 48–72 h poi. By contrast, the zfMR expression matched one-peak pattern in the intestine. It was significantly up-regulated at 3 h poi, then it was down-regulated significantly during 6–48 h poi.

### 2.5. Temporal Expression of IL-1β and TNF-α after the Infection with A. sobria

During bacterial infections, pro-inflammatory cytokines always involve in the innate immune responses. In order to obtain information about cytokines involved, IL-1β and TNF-α were chosen for study. As shown in Figure 4B, there were basically one-peak and two-peak expression patterns of IL-1β in the four tissues during infection. In the liver, spleen and intestine, the IL-1β expression fitted a one-peak pattern. In the liver, it was up-regulated significantly and reached a peak at 6 h poi. Thereafter, it was still up-regulated significantly until 48 h poi, and dropped to under the basal level at 72 h poi. In the spleen, the mRNA of IL-1β was up-regulated significantly and reached the peak at 3 h poi. Thereafter, it was still up-regulated significantly until 48 h poi, and was dropped to under the basal level at 72 h poi. In intestine, the mRNA of IL-1β were firstly down-regulated at 3 poi, then it was up-regulated significantly at 6 h poi, thereafter, it was down-regulated significantly during 12–72 h poi. In the kidney, the IL-1β mRNA expression fitted two-peak pattern. It was up-regulated significantly at 3 h poi, reached the first peak at 6 h poi. Thereafter, it was still up-regulated at 12 h poi, and dropped to the basal level at 24 h poi. However, it was up-regulated significantly again and reached the second peak at 48 h poi, eventually, it dropped to under the basal level again at 72 h poi.

TNF-α relative expression also showed one-peak and two-peak patterns (Figure 4C). In the spleen and intestine, the TNF-α mRNA expression fitted one-peak pattern. Regarding the spleen, it was significantly up-regulated and peaked at 3 h poi, and remained significantly up-regulated up to 48 h poi. Thereafter, its level was dropped below the basal level at 72 h poi. In intestine, the expression level of TNF-α after infection was significantly down-regulated at 3 h, up-regulated at 6 h, and down-regulated during the period from 12 to 72 h. In the liver and kidney, the TNF-α expression fitted a two-peak pattern. It was significantly up regulated as early as 3 h poi in the liver and 6 h poi in the kidney. Thereafter, its level dropped to the basal level during the period from 12 to 24 h poi, and then re-upregulated and peaked at 48 h poi in both organs.

### 2.6. The Protein Expressions of MR after A. sobria Infection

**Figure 5:**
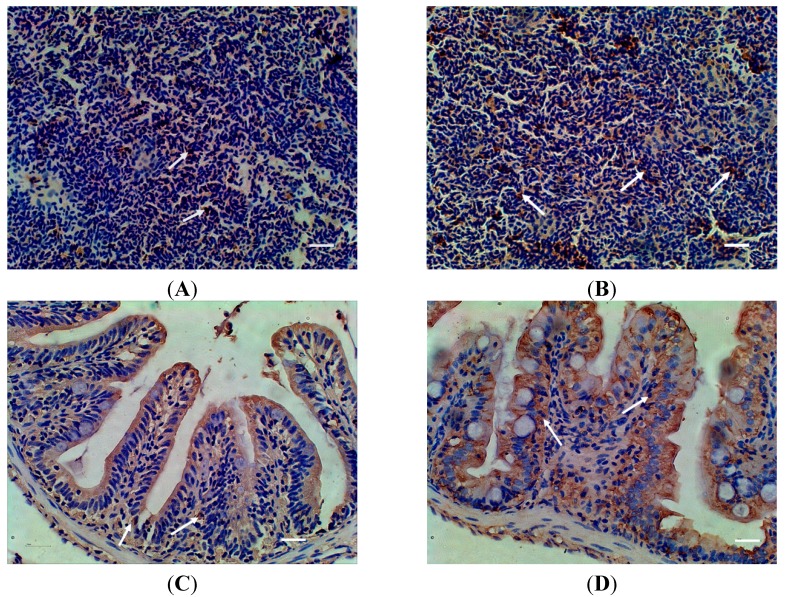
Distribution of MR in spleen and intestine of zebra fish detected with Immunohistochemistry after *Aeromonas sobria* infection. (**A**) spleen of control fish; (**B**) spleen of infected fish; (**C**) intestine of control fish; (**D**) intestine of infected fish; White arrows indicated positive signal. Scale bar = 20 μm.

To further monitor the MR expression at protein level during the infection, the expression levels of MR protein in different tissues were assessed by Western blot, the levels of MR protein between the infected and non-infected tissues were not significantly different (Supplementary Figure S2). Therefore, a more sensitive immunohistochemistry assay was employed in this study. The MR protein expression level after the infection with *A. sobria* was slightly increased in the spleen and intestine. No excess expression level was detected in other tissues (Figure 5).

## 3. Discussion

In the present study, we cloned, sequenced and analyzed the structure of zfMR. The deduced amino acid sequences of the zfMR shared highly conserved structures with MRs from the other species, indicating that zfMR should be a new member of C-type lectin superfamily. The existence of collagen binding sites and carbohydrate recognition sites in zfMR indicate that it might have collagen [2,30,31] and carbohydrate [28,29,32,33] binding activity. Furthermore, two conserved Ca^2+^ binding sites [2] were also found in zfMR, suggests that it might have calcium-dependent agglutination activity. All these activities need to be tested in the future. To evaluate the molecular evolution of the protein, MRs of nine animals from different taxa were used to construct a phylogenetic tree. The tree followed classical taxonomy, indicating that all MR proteins shared a single clade and they might have similar biological functions evolving from the same ancestor gene.

Revealing the mRNA expression profiles of MR, IL-1β and TNF-α in different tissues would help understand the mechanisms of MR in immune responses. The mRNA of MR was constitutively expressed in all tested tissues of non-infected in zebra fish. However, the higher expression levels of MR were observed in the kidney and spleen, indicating that it might play a role in the immune responses during the infection. After infection with *A. sobria*, the temporal expression of MR mRNA in liver, spleen, kidney and intestine were all significantly up-regulated at certain time points post infection. The reasons for the variation of the temporal expression patterns of zfMR in different tissues are enigmatic; however, the mRNA expression of zfMR was indeed induced by the infection of *A. sobria*, indicating that zfMR should play roles during the infection.

Strong inflammatory reactions were provoked by *A. sobria*, since the expression levels of IL-1β and TNF-α were up-regulated drastically in the liver, spleen, kidney and intestine which were immune related tissues. It has been shown that MR was involved in synthesis of pro-inflammatory cytokines such as IL-1β and TNF-α by binding of natural or synthetic ligands [34]. It is likely that the binding activity requires the assistance of other receptors in order to trigger signal cascades, since MR is lack of signal motifs in its cytoplasmic domain [1]. It has been reported that the release of IL-1β, TNF-α and IL-6 requires the collaboration of MR and TLR4 [35] and the IL-8 released by macrophages requires the expressions of MR and TLR2 [36]. In mammals, TLR4 is critical for the recognition of LPS and the release of pro-inflammatory cytokines [37,38]. It has been shown that fish TLR4 did not have the ability to identify LPS [39], however, it has been observed that TLR4 of *Gobiocypris rarus* could sense *A. hydrophila* infection and triggered signaling pathways [40]. It is well established that NF-κB plays a key role in immune and inflammatory responses and is a major mechanism of LPS-induced expression of cytokines, including IL-1, TNF-α and IL-8 [41]. The relationship between the expression of inflammatory cytokines and the involvement of zfMR needs to be elucidated. The establishment of a zebra fish-*A. sobria* infection model in this report will be valuable for further studies of the teleost MR functions during infection.

## 4. Experimental Section

### 4.1. Fish and Bacteria

The adult zebra fish were purchased from the Institute of Hydrobiology, Chinese Academy of Sciences, China. Zebra fish were 4~5 months old with total length of 3~5 cm. The fish were maintained at 25~26 °C in a re-circulating freshwater system and acclimatized in laboratory for at least one month before carrying out the experiments. *Aeromonas sobria* was isolated from a diseased zebra fish in our lab.

### 4.2. RNA Isolation and cDNA Synthesis

Trizol reagent (TaKaRa, Dalian, China) was used for RNA extraction according to the manufacturer’s instructions. The total RNAs were assessed with a NanoDrop Spectrophotometer (NanoDrop Technologies) and were stored at −80 °C until use. cDNAs were synthesized using PrimeScript™ RT reagent Kit with gDNA Eraser (TaKaRa) following the manufacturer’s protocol.

### 4.3. Entire MR cDNA Sequence by RACE

Pairs of primers (Table 1) were designed according to conserved sequences of MR genes from the predicted zebra fish MR gene sequence using primer premier 5.0 software and were used to amplify MR cDNA fragments. Total RNAs were isolated from the liver of zebra fish, subsequently, cDNA synthesis was performed and PCR reactions were carried out in a volume of 25 μL containing 12.5 μL of Premix Ex Taq (TaKaRa), 1 μL of 10 μM of each primer, 9.5 μL of nuclease-free water, and 1 μL of cDNA. Cycling parameters were 94 °C for 3 min followed by 35 cycles of 94 °C for 30 s, 58 °C for 30 s, 72 °C for 2 min, and final extension at 72 °C for 10 min followed by cooling down to 16 °C. The PCR products were resolved by electrophoresis on 1% agarose gel and the fragments of interest were excised, and then purified using the QIAEX II Gel Extraction Kit (Qiagen, Beijing, China). The purified fragments were ligated into pMD-19T vectors (Takara) and transformed into *E. coli* DH5α cells according to the standard protocol. Positive clones were screened and sequenced. Since it was difficult to obtain the full length MR cDNA sequences with one PCR amplification, overlapping partial sequences (Table 1) were amplified and combined using SeqMan of DNAStar. Based on the obtained MR fragment sequences, primers for 5'RACE and 3'RACE (Table 1) were designed. The RACE reactions were performed using a 5'RACE System for Rapid Amplification of cDNA Ends (Invitrogen, Shanghai, China) and a SMARTer™ RACE cDNA Amplification Kit (Clontech, Beijing, China) according to the manufacturer’s protocols.

**Table 1:** Primers used in the experiments.

Names	Sequence (5'→3')	Amplification Target
5'1	CATTTGCTTCTGATTCC	5'RACE
5'2	CAGCCAGGAACAGAGTCTCA	5'RACE
5'3	GTTCCCATGGCATTCCCACT	5'RACE
ZF1	CGCATTTTGAACACTTTCACAA	cDNA fragment of MR
ZR1	TGCTCCACTGCCATCCACTG	cDNA fragment of MR
ZF2	CAGTGGATGGCAGTGGAGCA	cDNA fragment of MR
ZR2	TCCAGAAGTACTTCTCTGGTC	cDNA fragment of MR
ZF3	GACCAGAGAAGTACTTCTGGA	cDNA fragment of MR
ZR3	CTGTTGAGACCAATCCACATAGGCT	cDNA fragment of MR
ZF4	AGCCTATGTGGATTGGTCTCAACAG	cDNA fragment of MR
ZR4	TGATGATCCCTGCCATTGTTTA	cDNA fragment of MR
ZF5	ACCACAGTTGATGAACCGGA	cDNA fragment of MR
ZR5	GCCATGTTTGCTTAGTGCGG	cDNA fragment of MR
ZF6	ATGCCGAAGACTGCGTGTTA	cDNA fragment of MR
ZR6	CCAGGGCTTGATCCCATGTT	cDNA fragment of MR
ZF7	TCGTCGCTGTAAGGCAGAAG	cDNA fragment of MR
ZR7	CGGTCTCAGTTAGTGCCCAG	cDNA fragment of MR
3'1	GCAAAACCGCCAAAGTGATTAGCCCA	3'RACE
3'2	GGTCACAACATCGCTTCCTGCCTACA	3'RACE
β-actin-F (forward)	ATGGATGAGGAAATCGCTG	β-actin Expression
β-actin-R (reverse)	ATGCCAACCATCACTCCCTG	β-actin Expression
MR-F (forward)	GGGACCTATTATGCCCCTCT	MR Expression
MR-R (reverse)	AGGCATTTGTAGCTCTGCACT	MR Expression
IL-1β-F (forward)	GGACTTCGCAGCACAAAATGA	IL-1β Expression
IL-1β-R (reverse)	GACGGCACTGAATCCACCAC	IL-1β Expression
TNF-α-F (forward)	CTGAGGAACAAGTGCTTATGA	TNF-α Expression
TNF-α-R(reverse)	GTAGAAGTGCTGTGGTCGTG	TNF-α Expression

### 4.4. Sequence Analysis

The MR amino acid sequences from various species were obtained from NCBI. The cDNA and deduced amino acid sequence of MR were analyzed using the BLAST algorithm and the Expert Protein Analysis System (http://www.expasy.org/). Multiple sequence alignment was performed with the DNAMAN program. A phylogenetic tree was constructed using the neighbor-joining method in the Molecular Evolutionary Genetics Analysis package (MEGA 4.0, Tokyo, Japan). Data were analyzed using Poisson correction, and gaps were removed by complete deletion. The topological stability of the trees was evaluated by 1000 bootstrap replications.

### 4.5. Quantitative Real-Time PCR (qRT-PCR) Analysis of MR mRNA Expression in Different Tissues

The total RNAs from liver, spleen, kidney, intestine, gill, brain, heart, muscle and skin were extracted from fifteen fish and quantitative real-time PCR (qRT-PCR) was performed to determine MR mRNA expression. The size of the zebra fish was too small to differentiate between the head kidney and body kidney. Therefore, we have utilized the whole kidney to represent both kidney parts. Briefly, the cDNA was synthesized as mentioned above, diluted to 1:10 and stored at −20 °C for subsequent qRT-PCR. β-actin was used as an internal control. Primers used for qRT-PCR were shown in Table 1. The assay was conducted on Rotor-Gene Q Series Real-Time PCR System (Roche Molecular Systems, Belleville, NJ, USA) in a final volume of 20 μL containing 1 μL cDNA sample, 10 μL SYBR^®^*Premix Ex Taq*™, 1 μL of each forward and reverse primers (10 μM) and 7 μL nuclease-free water. Cycling parameters were 95 °C for 30 s followed by 40 cycles of 95 °C for 5 s, 58 °C for 20 s, 72 °C for 20 s, 4 °C for 5 min. Dissociation curve analysis was performed to determine the target specificity. The relative expression ratio of the target gene *versus* the β-actin gene was calculated using 2^−ΔΔ*C*t^ method. All data were given in terms of relative mRNA expressed as mean ± SE (*n =* 3).

### 4.6. The mRNA Expressions of MR, IL-1β and TNF-α after A. sobria Infection

Challenge experiments were conducted as described previously with slight modification [40]. In brief, *A. sobria* was grown in the Luria-Bertani (LB) nutrient agar, and then diluted with 1× PBS to reach the density of 1.0 × 10^7^ CFU/mL. Three hundreds fish were randomly divided into 2 groups. Fish were anaesthetized by immersion in MS222 and injected intraperitoneally (IP) with 20 μL bacterial suspension whereas control fish were injected equal volume of PBS. Kidney, intestine, liver and spleen were collected from each group at 3, 6, 12, 24, 48 and 72 h post of the infection (poi). Tissues from fifteen fish were frozen in liquid nitrogen immediately after collection, and stored at −80 °C until use. RNA isolation, cDNA synthesis and qRT-PCR were carried out as described above. The relative expression ratio of the target gene *versus* the β-actin gene was calculated using 2^−ΔΔ*C*t^ method. All data were given in terms of relative mRNA expressed as mean ± SE (*n* = 3). The data were submitted to one-way analysis of variance (one-way ANOVA) followed by Fisher’s LSD test using SPSS 17.0. Differences were considered significant at *p* < 0.05 and extremely significant at *p* < 0.01.

### 4.7. The Expression of MR Protein in Different Tissues of Zebra Fish after Infection with A. sobria

Immunohistochemistry (IHC) was performed to observe the expression of MR in the tissues of zebra fish. Fresh tissues were fixed with 4% paraformaldehyde prior to the tissues were embedded and sectioned using the standard method. The sections were incubated with 100% methanol containing 3% H_2_O_2_ at room temperature for 10 min to block endogenous peroxidase activity. After washing with PBS, the slides were incubated in citrate buffer (1.8 mM citric acid, 8.2 mM sodium citrate) at 95–100 °C for 10 min to unmask the antigenic epitope. The sections were incubated with blocking buffer (10% bovine serum albumin in PBS) at room temperature for 1 h. Thereafter, the sections were incubated with MR rabbit specific antibody generated in our lab at the dilution of 1:1000 at room temperature for 1 h. After washing with PBS, the slides were incubated with anti-rabbit IgG-HRP for 30 min and revealed in DAB solution (Guge Biology, Wuhan, China).

## Supplementary Materials

Supplementary materials can be found at http://www.mdpi.com/1422-0067/16/05/10997/s1.
