# Supplementary material for: Molecular Cloning and Functional Characterization of Mannose Receptor in Zebra Fish (Danio rerio) during Infection with Aeromonas sobria
Source: Int J Mol Sci. 2015 May 15;16(5):10997–1012. doi: 10.3390/ijms160510997 (PMC4463687; doi:10.3390/ijms160510997)
Supplement: Supplementary file 1 [file ijms-16-10997-s001.pdf]

Supplementary Information

1	GGGGGGGGGCCGTGTATCCTGACGTGATTCCCGCGGAGTCTGCAGAAGCTCAGCCAAAAG
61	GCAGAGGATAATGCATAAAAGAGCAGCACTTTGCATTTAGGATTACATTTAGAATCAAG**A**
121	**TG**AAAATAATTACCGTGGTCGTTTTGCCTGTATTGGAGCTGTCAAACTGCTTTGCACAAT
1	M K I I T V V V L P V L E L S N C F A Q
181	CAGGAGACAGCTTTTTATTATACAATGTGGACTCTAATAAATGCCTGACTAGCACTTTGA
21	S G D S F L L Y N V D S N K C L T S T L
241	ACAGGCTTGTCACCTGTGACCCACACAGTGCCCAACAAAAGTTCCGCTGGACTTCAAGTA
41	N R L V T C D P H S A Q Q K F R W T S S
301	ACCGCATTTTGAACACTTTCACAAAGACGTGTCTTGGAGTGGGAAGTAAAGCGGTGGGCA
61	N R I L N T F T K T C L G V G S K A V G
361	AAACTCTGCAGCCCTTAAAGTGCAACGATGACAATGCTCTGCAGAAGTGGGAATGCCATG
81	K T L Q P L K C N D D N A L Q K W E C H
421	GGAACACATTGCTTGGACTAAAGAATGAGACTCTGTTCCTGGCTGTAGATTTTAGGGGTT
101	G N T L L G L K N E T L F L A V D F R G
481	TACCTGAGATTTCTAATAAGACTGGAATCAGAAGCAAATGGACAATTCATGGCACACAGG
121	L P E I S N K T G I R S K W T I H G T Q
541	ACAGCATTTGCTCTCGGCCTTATGAAGAAATCTACAGCATTGATGGAAATGGATTTGGGC
141	D S I C S R P Y E E I Y S I D G N G F G
601	AGACATGCAAGTTTCCTTTTTTGTATGAGAAAAAGTGGTATGCAGATTGTACCACAGTTG
161	Q T C K F P F L Y E K K W Y A D C T T V
661	ATGAACCGGACCAGCGTCTGTGGTGCGCAACCAAGACTGACTACAGTTTATATGAGCAGT
181	D E P D Q R L W C A T K T D Y S L Y E Q
721	GGGGATACTGTCCAACACGTGATAGTAAATACTGGACAAAACATCCTCTAACAAACGTCT
201	W G Y C P T R D S K Y W T K H P L T N V
781	ACTACCAGCTGAATGACAGGTCAACTCTGACATGGTACCAGGCTAGAAAGAGCTGTCAGC
221	Y Y Q L N D R S T L T W Y Q A R K S C Q
841	AGCAAGGCGCTGAATTGCTGAGCATATCTGAACCTCACGAACAATCCTTCATAGCAGGAA
241	Q Q G A E L L S I S E P H E Q S F I A G
901	TGTTTCAGAAGTCACAAGGCTCACTATGGATAGGACTGAACAAGTTAGATGTGTCCAGTG
261	M F Q K S Q G S L W I G L N K L D V S S
961	GATGGCAGTGGAGCAATGGACAGCCTTTACGCTATTTGAAATGGCTCAGTGGATTCCCAA
281	G W Q W S N G Q P L R Y L K W L S G F P
1021	GCTCACAACCAGGCTACAATTGTGGCGTCTTGAAAAATGGCTATAATTCAGAATGGTCAA
301	S S Q P G Y N C G V L K N G Y N S E W S
1081	ATGATGCTTGCTCTGAAAAACGTGGATACATCTGCCAACGAGGTCATTCTGTTCCTACTG
321	N D A C S E K R G Y I C Q R G H S V P T
1141	TTCCACCAGAAGTGACGACTGGATTTTGCCAAAGCCCCTGGATTCCACATTCTGGCAACT
341	V P P E V T T G F C Q S P W I P H S G N
1201	GTTATTTTCTACACCGCACTAAGCAAACATGGCTGGAGGCACGGGACATCTGTCTGCGGG
361	C Y F L H R T K Q T W L E A R D I C L R
1261	AAGGAGGAGACCTGCTAAGTATTCTCAGCACAGAAGAGCAAAGCTTTGCCATCACACAGC
381	E G G D L L S I L S T E E Q S F A I T Q
1321	TTGGATACTCAAAGACTGATCAGCTGTGGATTGGTTTCAATGACCGCAAAACACAGATGT
401	L G Y S K T D Q L W I G F N D R K T Q M

Figure S1. *Cont.*

```
1381    TGTTTGAATGGAGTGACCAGTCTAGCGTCCCGTTTGCCTCATGGGAGGTTGGCGAGCCGA
421     L  F  E  W  S  D  Q  S  S  V  P  F  A  S  W  E  V  G  E  P
1441    CTCACAGTGCTCAGCATGCCGAAGACTGCGTGTTAATGAGAGGGGAGGAGGGAAAGTGGG
441     T  H  S  A  Q  H  A  E  D  C  V  L  M  R  G  E  E  G  K  W
1501    CTGATGATGTTTGTGAAAAAAAATATGGCTTCATCTGTAAGAGAAAGACCAGCACTAAAG
461     A  D  D  V  C  E  K  K  Y  G  F  I  C  K  R  K  T  S  T  K
1561    CCTCAAATAATGACACGGTTGTCGCAAATCCAGGATGCAAAAAGGGCTGGATCAGGTATG
481     A  S  N  N  D  T  V  V  A  N  P  G  C  K  K  G  W  I  R  Y
1621    GGTACTATTGTTACATGGCAGGATCCGAGACAAAGACCTCCGAAGAGGCAAAACAGACGT
501     G  Y  Y  C  Y  M  A  G  S  E  T  K  T  S  E  E  A  K  Q  T
1681    GTGAAAAAGCAGAGTCTCGACTTGTAGATGTTTCATCCAGAGTAGAAAATGCATTCCTGG
521     C  E  K  A  E  S  R  L  V  D  V  S  S  R  V  E  N  A  F  L
1741    TTAATCTAGTAGGAGCACGACCAGAGAAGTACTTCTGGATTGGACTGTCTAATCAGAAGG
541     V  N  L  V  G  A  R  P  E  K  Y  F  W  I  G  L  S  N  Q  K
1801    ACGTACACACTTTTGAGTGGACCAACACTAAGCAAGTCCCATTCACTCACTTCAACTCTG
561     D  V  H  T  F  E  W  T  N  T  K  Q  V  P  F  T  H  F  N  S
1861    GGATGCCAGGAAGAAAACAAGGCTGTGTTGCAATGACGACTGGAATAGTTGCTGGGCTTT
581     G  M  P  G  R  K  Q  G  C  V  A  M  T  T  G  I  V  A  G  L
1921    GGGATGTGCTTAGCTGTTCAAATAAGGAAAAATACATCTGCAAGCAAAGAGCTGATGCTC
601     W  D  V  L  S  C  S  N  K  E  K  Y  I  C  K  Q  R  A  D  A
1981    TAGTAACAACCGCAGCCCCGCCAACCACCCCTTCCCTGGACTGTCCCACAGAATGGACTT
621     L  V  T  T  A  A  P  P  T  T  P  S  L  D  C  P  T  E  W  T
2041    CAATTGGGACAAGAGACCTCTGTGTCAAGCATTTCAATGTACCTTCACTGCAAATGAAAA
641     S  I  G  T  R  D  L  C  V  K  H  F  N  V  P  S  L  Q  M  K
2101    CATGGGATCAAGCCCTGGACTACTGCAGAGAACTCGGTGGTGACCTCCTGAGCATCCATC
661     T  W  D  Q  A  L  D  Y  C  R  E  L  G  G  D  L  L  S  I  H
2161    ATGAATCTGATATTCCCTGGAAACAAGGAGGAGGGTATCCATCTTGGATTGGTTACAGAA
681     H  E  S  D  I  P  W  K  Q  G  G  G  Y  P  S  W  I  G  Y  R
2221    TGTATGATCCCTCTGTGGGTTACGTTTGGAGTGACGGCTCTTCGTCGTCCTATCAAAGCT
701     M  Y  D  P  S  V  G  Y  V  W  S  D  G  S  S  S  S  Y  Q  S
2281    GGGCCAGCGATGAACCAAACAACCTAAACAACATGGAAAATTGTGTTGAAATGAGAGTGT
721     W  A  S  D  E  P  N  N  L  N  N  M  E  N  C  V  E  M  R  V
2341    CGCTGTGGGACGATGATGGGATGTGGAATGACGTGAACTGTAAAGACAAGAAGGACTGGT
741     S  L  W  D  D  D  G  M  W  N  D  V  N  C  K  D  K  K  D  W
2401    ACTGTCAGATCCACAAAGGAAAGACTCCAGTTGAGGTGAATATTACAGAACCAGTTTATA
761     Y  C  Q  I  H  K  G  K  T  P  V  E  V  N  I  T  E  P  V  Y
2461    ATGTAACAGAGGATGGCTGGATTGAATTCAGAGGTAGCCAGTATTATGGGTCCGAGTACT
781     N  V  T  E  D  G  W  I  E  F  R  G  S  Q  Y  Y  G  S  E  Y
2521    CAGCGATGTCTATGCATGAAGCACGGGCGTTCTGTAAAAGAAATCATGGCGATCTTGTAG
801     S  A  M  S  M  H  E  A  R  A  F  C  K  R  N  H  G  D  L  V
2581    TCATCAACGATGAGGAGGAGCGACTGTTCCTCTGGCATAAGTCTAAAGAGTTGTACAACG
821     V  I  N  D  E  E  E  R  L  F  L  W  H  K  S  K  E  L  Y  N
2641    ATTTTCTCATTGGCTTGACGGTTGATCTGGATGGATCTTTCCAGTGGATGGATGGGTCTC
841     D  F  L  I  G  L  T  V  D  L  D  G  S  F  Q  W  M  D  G  S
```

Figure S1. *Cont.*

2701 CTGTTGTGTTTCAAGCTTGGGAAGCAAATCAACCTGCCTTTAAAAACAGTGAGGAAAGGT
861 P V V F Q A W E A N Q P A F K N S E E R
2761 GCGCAAAGATGACCATATCTCAAGGACTCTGGGAAACCGTCAACTGTGGTGATGAATATA
881 C A K M T I S Q G L W E T V N C G D E Y
2821 ATTATTTTTGCAAGCGAAGTGAGGCTCCTCCAGTTAATGCTACTGTGGCCCCTACACAGC
901 N Y F C K R S E A P P V N A T V A P T Q
2881 CACCAAAAGGAGGCTGTGCGCCTGAGTGGACACAGTTCGAGGGAAAGTGCTACAATGTGA
921 P P K G G C A P E W T Q F E G K C Y N V
2941 GGGGGGAAATGAAAAAATGGAGCGAAGCAAGAGAATACTGCAGAGAACACGGTGGAGATC
941 R G E M K K W S E A R E Y C R E H G G D
3001 TGACAGCTATTATGAGCAAATTCCAGCAAACATTTTTAAGCACAATGATTCGAGATAAAT
961 L T A I M S K F Q Q T F L S T M I R D K
3061 CCACTAACTTTTGGATTGGATTCAGCAATCTGGCAAATGGAAGGTTCAAGTGGACAGATG
981 S T N F W I G F S N L A N G R F K W T D
3121 GGAGTAAAGTTTCATTCACAGAGTGGGCTGAAGGGGAACCTCATTCCTTAGTATGGTCAC
1001 G S K V S F T E W A E G E P H S L V W S
3181 GTTCATACTACTGGACAAAATACTTTTCGGACGAGCCGGAATGTGTTTTTATGGGCAGGA
1021 R S Y Y W T K Y F S D E P E C V F M G R
3241 GTTCAGGATCTCACTTTAGCAAGTGGGTGGCAGACGACTGTAATTCTACTAATGGCTTCA
1041 S S G S H F S K W V A D D C N S T N G F
3301 TTTGCAGTCGTGATGTTGATCCAGGTATCCCCTCAGTGCCAACTGAGATTCCTAAAACCT
1061 I C S R D V D P G I P S V P T E I P K T
3361 TTGTCAAGCTTGGAAATTCATCTTTCAAAGTGATTCAAGAAAACCTAACGTGGATTGAGG
1081 F V K L G N S S F K V I Q E N L T W I E
3421 CAAATCGTCGCTGTAAGGCAGAAGGGGGTCATCTGGCCAGTATTCGGGACTTGATATCAC
1101 A N R R C K A E G G H L A S I R D L I S
3481 AAGCTTACATTGAGTTGCAGGTCTTCAGACTCAAGCAGCCTATGTGGATTGGTCTCAACA
1121 Q A Y I E L Q V F R L K Q P M W I G L N
3541 GTGAACAGTCAAATGGATATTTTCTGTGGGTGAATAAATGGCCAATGACCATGGAGAAAT
1141 S E Q S N G Y F L W V N K W P M T M E K
3601 GGGCGATATCTGAACCTAGGCCCAACAAACCTTGTGCACACATGAAAATAAATGGAGAAT
1161 W A I S E P R P N K P C A H M K I N G E
3661 GGAAAACGTCTCTATGCAATGAAACCTTCTACAGTGTCTGTGAGCAAACAACGGACATTC
1181 W K T S L C N E T F Y S V C E Q T T D I
3721 CGCCAACCCTTCCAGCACAGCAGCCCGGACACTGTCCAAAGCAAGAAAATTACAGTCCCC
1201 P P T L P A Q Q P G H C P K Q E N Y S P
3781 TGAGGTGGATACCTTTCAGAGACAGCTGCTATGCTTTTGTGACAGAAATGAAATCATGGA
1221 L R W I P F R D S C Y A F V T E M K S W
3841 GCAGAGCAGCAAGACTTTGTATGACATGGGGAGCTTCTCTTGCCAGCATCAGAGATGAAG
1241 S R A A R L C M T W G A S L A S I R D E
3901 CGGAGGAGAAGTTTATAGAAAGCAACCTCTTGCTCCTTGAAAGTTATAAAGAATTTTGGA
1261 A E E K F I E S N L L L L E S Y K E F W
3961 TTGGATTGTTACACAACCATAAAGGACACTGGTTATGGGCAGACAACAGTGTGGTAGATT
1281 I G L L H N H K G H W L W A D N S V V D

Figure S1. *Cont.*

4021 ACACTAACTGGGCACTAACTGAGACCGGTGGTGACTATGAAGAATTTTCTTATAATCCTG
1301 Y T N W A L T E T G G D Y E E F S Y N P
4081 ACTGCGCATTTATCTCTGCCACGTCTAAAAATTGGAAAAAACGCCACTGTGATTACACAG
1321 D C A F I S A T S K N W K K R H C D Y T
4141 TTTTACCGTTCATCTGCAAAACCGCCAAAGTGATTAGCCCAACTATTAAGTCCCCACATC
1341 V L P F I C K T A K V I S P T I K S P H
4201 AAGATATTAAACCTCATAGGGTTAAAACTGGATTGGCTGTATTCTTGACTATTGCAGTGA
1361 Q D I K P H R V K T G L A V F L T I A V
4261 TTGCCATACTTGGAGCTCTAGCCTATGTTTACTACAGAAGGTCACAACATCGCTTCCTGC
1381 I A I L G A L A Y V Y Y R R S Q H R F L
4321 CTACATTTGAAAATCCCATGTATAATAACGCAGAGTCTGCTTATTCAGATAAAGATAACA
1401 P T F E N P M Y N N A E S A Y S D K D N
4381 AATCATTGCTTGAGGTTGCAGAA**TAG**ACCTGTACATGTAGTCATCTGGCCAGTATCATAA
1421 K S L L E V A E *
4441 AATAAACAATGGCAGGGATCATCAAGTTTATTTTGTGATATCGGCTCCCTACTTCTTTTA
4501 CATGGCTGGATGCCAAATAAATAATTGATTTGGTTAGAAAAGGAACGTAGCCATGTAAAT
4561 CAGTTGTC**ATTTA**AAGAGGGACCTATTATGCCCCTCTTTACAAG**ATTTA**AAATAAGTCTC
4621 TGATGTCTCTAGAGCGTGTATGTGAAGTTTATCCCAAAATACCTCACCAATAATG**ATTT**
4681 **A**TTACTGCCCCTTCTAGGCTTTGATCCTAACTGTGTTATTTTGGTGACTGTTGCTTTAAG
4741 TTGAAATAAGATTGTGCTTTTAGCCCTCTTTTCAAAAGAGTGCAGAGCTACAAATGCCTT
4801 TGTGTCAGCATAGTGGCAGATTCAAAAACAGGACTAACTTCTTACGCTAATGAGGGAGAC
4861 ATCATCATTAATAGGTGGGGCTTTCCCCTTCATGTACAAAGGGAGAATGTCAATCAAAGT
4921 ATTTGTGTAGAACTTTTTGAAGTGTGATTAGAAAAATTAAAGTTATTTTTTACCATTAGA
4981 AACGGGTTATATTCACACAGTCGCCACACAACTGTTTAAACCCCTTATAAAGGTGATTTT
5041 TGCATAATGGATCCCCTTTAATTAGTTTAAAAGTCATTATGATCAAATAAATAATAATAA
5101 AATCTGACTGCACAATGGTGCAACTGGCTAATCATATCATACTACAATTGCTGGTTTTAA
5161 TTATTTTCTCCAAAAGCTTTTGAACATCAGACTTTGTTATTAAATGTTAAAGTTCAAAAA
5221 TAAATGTCTCTAATAACCTTCAGTTTTTACACTATGGTATATTTGCATTGACTGGATTAA
5281 CACTCACAATGCAAAAAGTTTTAAGGAAGGAAACAATGTCAACTTTTTTTGTGCCAAAAA
5341 ATTGTGGATGTAAGGTCAGCTGACACTTGAAAGCTGACAGAGTTAGCATTTGGAGAGACT
5401 TGTGCAAATGTTAT**ATTTA**TACTGTAAATGAACACTCCAAAAGTTCTTTATATATTCATA
5461 TTCTATGACTAATCCATAATGAATACAGAATAGGGGACTTTGGGTGGGAACGATTCCGAA
5521 AATCACCAACTGATAAATGTGATGTGTGCATTATTGGTTTAGGAGAGCATTTCAATAACA
5581 ACAATATACAGTCAGATCAACTGAATACAGAAAGAACATCTATATTACAGTGCAGCAGTA
5641 TCTGTACTCAGATTCATTAATCCAATATCTACAAAGGC**ATTTA**TGAGGTATATACAAATG
5701 CATAGAAACAATTTTCAAGCTATAGTGTTGCCACAAACTAATGCATAGAGTACTCAGGAA
5761 ACATACAGGTATCAAATGTAAATAAAAGATATTGTTTCAGATGCATTGAGGATATGATAT
5821 TGCCATCTTTTGATTAACAGTTTCAACAAAAGTGAAAACAAAAC**ATTTA**CCATGCTGTTG
5881 TAATGAAACAAGTATTGTACCTATTTTTGTCAGTATATTTGTGTGTCTTTTAACTTTATG
5941 AAGATAAAAGTAAAAAAAAAAAAAATCATACATACAGGATTGTACATATTTCAATAAACAT
6001 TCTGAAAATCATAAATACATGCATATGGTTGGCTAAAAATGATCACGTTGTATATATTAT
6061 TAATATGGAAATACAGAACCTAAAACATCAACAGTGTTCATTTGCAATAACTTTGCATGA
6121 GCAAAGTACACATAATATGGCTCGAGAGTTG**ATTTA**CATGATTGTGGTCTTTGTATTGAC
6181 AAAAATGATTCTTTATT***AATAAA***CAGGAAATTGAATATCTTAAAAAAAAAAAAAAAAAA
6241 AAAAAAAA

Figure S1. Nucleotide and deduced amino acid sequences of MR from *Danio rerio*. The start codon (ATG) was shown by bold letters. The termination code (TAG) was shown by bold letters and marked with an asterisk below, and the putative signal peptide was underlined. The motif associated with mRNA instability (ATTTA) was in bold. The polyadenylation signal (AATAAA) was tagged with bold italic.

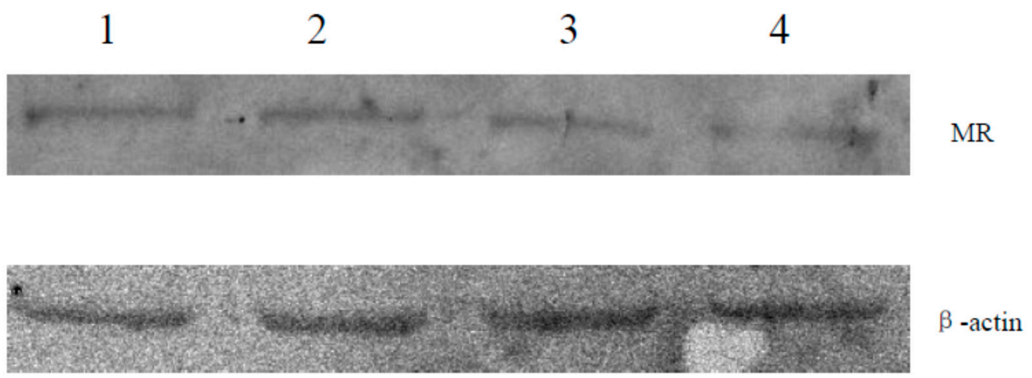

Figure S2. The expression of MR in the liver and spleen of zebra fish with or without the infection of *Aeromonas sobria*. The upper part of the figure is MR, the lower part of the figure is β-actin. Lane: 1: liver of control fish; 2: liver of infected fish; 3: spleen of control fish; 4: spleen of infected fish.
